# Symposium Introduction

**DOI:** 10.2174/157340312803217256

**Published:** 2012-08

**Authors:** John A Ambrose, MD

For the interventionalist, intracoronary thrombus remains an important marker for the presence of an acute coronary syndrome as well as a harbinger of potential complications during the percutaneous procedure. Acute complications include myocardial infarction either new and/or recurrent related to coronary embolization, slow coronary flow or acute closure and long term complications include sub acute and late stent thrombosis and possibly restenosis. Over the years, there have been several advances in the development of new and more potent antithrombotic and anticoagulant therapies as well as new interventional and pharmacologic approaches for dealing with intracoronary thrombus before, during and immediately after percutaneous intervention.

The purpose of this symposium is at least three fold: 1. Define the pathophysiology of intracoronary thrombus and its relationship to acute and long term complications. 2. Describe the best antithrombotic and anticoagulant approaches in STEMI and NSTEMI. 3. Review the mechanical and pharmacological approaches available to the interventionalist for thrombus removal or/dissolution and/or treating its complications. All of these approaches and particularly for the antithrombotic and anticoagulant therapies must be balanced by the potential for side effects especially the possibility of increasing bleeding complications. I hope this symposium will provide you with important information as well as stimulate further investigation in this area.

